# What do we Know about Complex-Contrast Training? A Systematic Scoping Review

**DOI:** 10.1186/s40798-024-00771-z

**Published:** 2024-09-27

**Authors:** Rohit K. Thapa, Anthony Weldon, Tomás T. Freitas, Daniel Boullosa, José Afonso, Urs Granacher, Rodrigo Ramirez-Campillo

**Affiliations:** 1 Symbiosis School of Sports Sciences, Symbiosis International (Deemed University), Pune, 412115 India; 2 Centre for Life and Sport Sciences (CLaSS), Faculty of Health, Education and Life Sciences, Birmingham City University, Birmingham, B15 3TN UK; 3 Aston Villa Foundation, Aston Villa Football Club, Birmingham, B6 6HD UK; 4 UCAM Research Center for High Performance Sport, UCAM Universidad Católica de Murcia, Murcia, Spain; 5 Facultad de Deporte, UCAM Universidad Católica de Murcia, Murcia, Spain; 6 NAR—Nucleus of High Performance in Sport, São Paulo, Brazil; 7 Faculty of Physical Activity and Sports Sciences, Universidad de León, León, Spain; 8 Integrated Institute of Health, Federal University of Mato Grosso do Sul, Campo Grande, Brazil; 9 College of Healthcare Sciences, James Cook University, Townsville, Australia; 10 Centre of Research, Education, Innovation, and Intervention in Sport (CIFI 2 D), Faculty of Sport, University of Porto, Porto, Portugal; 11 Department of Sport and Sport Science, Exercise and Human Movement Science, University of Freiburg, Sandfangweg 4, 79102 Freiburg, Germany; 12 Exercise and Rehabilitation Sciences Institute, School of Physical Therapy, Faculty of Rehabilitation Sciences, Universidad Andres Bello, Santiago, 7591538 Chile

**Keywords:** Plyometric Exercise, Human Physical Conditioning, Muscle Strength, Team Sports, Exercise, Physical Fitness

## Abstract

**Background:**

The complex-contrast training (CCT) method utilizes two exercises with different loads and movement velocities in a set-by-set fashion to induce multiple neuromuscular adaptations. The speculated primary mechanism involves the post-activation potentiation or post-activation performance enhancement (PAPE) of the muscles used during the heavy load (low velocity) exercise, thereby improving the performance of lower load (high velocity) exercise. However, no previous study has attempted to systematically synthesize the available evidence on CCT (e.g., if post-activation potentiation or PAPE was measured during the training sessions during the intervention period). This study aimed to synthesize the available evidence on CCT using a systematic scoping review approach. More specifically, we identified gaps in the literature using an evidence gap map (EGM), and provided future directions for research.

**Methods:**

Three electronic databases (PubMed, Scopus, and Web of Science) were searched up to 20th February 2024. Data were extracted under a PICO framework: (a) Participants-related data (e.g., age, sex, type of sport); (b) Intervention-related data (e.g., duration of training); (c) Comparators (when available); and (d) Outcomes (e.g., measures of physical fitness). Interactive EGMs were created using the EPPI mapper software.

**Results:**

From the 5,695 records screened, 68 studies were eligible for inclusion, involving 1,821 participants (only 145 females from 5 studies). All CCT interventions lasted ≤ 16 weeks. More than half of the studies assessed countermovement jump, sprint, and maximal strength performances. No studies were identified which examined upper-body CCT exercises alone, and no study assessed PAPE during the CCT sessions. Overall, the available evidence was rated with a low level of confidence.

**Conclusions:**

In conclusion, whether CCT produces a PAPE that translates into longitudinal performance gains remains unclear. Moreover, the available evidence on the effects of CCT on various outcomes provides low confidence regarding the most effective way to implement this training method, particularly among females, and beyond long-term interventions.

**Supplementary Information:**

The online version contains supplementary material available at 10.1186/s40798-024-00771-z.

## Key Points

The current evidence of complex-contrast training (CCT) for various outcome variables (e.g., sprinting, jumping) includes a limited number of participants (*N* < 800).

Longitudinal studies beyond sixteen weeks of duration were unavailable, and only a small number of studies (*n* = 5/68) were available on female participants.

Previous studies have considered the post-activation performance enhancement (PAPE) phenomenon during CCT sessions as one of the main mechanisms responsible for improving physical fitness attributes (e.g., sprinting, jumping). However, no longitudinal study has assessed PAPE responses during CCT sessions. Hence, the role of PAPE in physical fitness improvement after CCT has yet to be clarified.

**Supplementary Information:**

The online version contains supplementary material available at 10.1186/s40798-024-00771-z.

## Background

Enhancing athletic performance is possible using various resistance training methods across the force-velocity continuum that target multiple neuromuscular adaptations [1]. For instance, heavy resistance training primarily targets the development of maximal strength [2]. Conversely, ballistic resistance training involves explosively projecting an individual’s body or an external load into the flight phase [3]. Ballistic exercises can be performed without external load (e.g., body mass plyometric) or with low loads (e.g., 30% one-repetition maximum [1RM]), primarily targeting the development of high-velocity movements, rate of force or torque development^1^ [4]. Therefore, depending on the loads and velocities attained during both training strategies, improvements can be expected across the force-velocity spectrum^2^ [5]. Therefore, to holistically develop an athlete’s force-velocity capabilities (i.e., strength and speed), utilizing a combination of heavy (slower) and lighter (faster) resistance training methods may be sought [6]. However, training both resistance training methods on separate days (i.e., compound training) or conducting sessions in sports with congested fixture schedules may be time-consuming and impractical [6, 7]. Nonetheless, the combination of both methods within a single training session (i.e., complex training) [5] can allow a time-efficient approach to improve athletes’ performance [8, 9].

A more detailed analysis of the scientific literature [5] reveals that complex training has been applied using four different combinations: (i) complex-descending, i.e., high-load exercise sets followed by low-load exercise sets (e.g., three heavy squat sets completed before three standing broad jump sets) [10], (ii) complex-ascending, i.e., low-load exercise sets followed by high-load exercise sets (e.g., three standing broad jump sets completed before three heavy squat sets), (iii) complex-contrast (CCT), i.e., heavy-load and low-load exercises in a set-by-set fashion (e.g., one heavy squat set followed by one set of standing broad jumps]) [11], and (iv) French-contrast training, i.e., subset of contrast training in which a high-load exercise is followed by a low-load exercise, followed by a low to moderate load exercise, followed by a low-load exercise (e.g., heavy squat, followed by standing broad jumps, followed by loaded jump squat, followed by band assisted countermovement jump [CMJ]). Amongst these sequencing methods, CCT is of further interest as it involves performing high-load and low-load exercises in alternating sequence that might result in post-activation performance enhancement (PAPE) of the latter exercise. In a systematic review with meta-analysis [12], when CCT was compared to other training methods (e.g., complex-descending), despite non-significant between-group differences, CCT led to larger effect sizes (ES) improvements in maximal strength (ES = 2.01 vs. 1.29), vertical jump performance (ES = 0.88 vs. 0.55), linear sprint performance (ES = -0.94 vs. -0.27), and change of direction speed (ES = -1.17 vs. -0.68) [12]. However, the above described meta-analysis [12] involved a comparison of within-group analysis, which should be interpreted with caution [13].

If CCT improves performance, two main mechanisms may be involved. First, the inclusion of exercises from both ends of the force-velocity spectrum may improve performance (e.g., sprinting, jumping) by allowing athletes to produce higher forces during faster movement velocities [4, 6, 7, 14]. Second, the performance of a heavy-load exercise might enhance the force and (contraction) velocity in the subsequent lower-load exercise by taking advantage of the PAPE phenomenon [15]. However, while PAPE effects have primarily been shown in cross-sectional studies, less is known about whether these acute PAPE effects translate into chronic adaptations. Several meta-analyses [12, 16–19] and reviews [5, 7, 20, 21] have recently been published regarding the effects of CCT as a potential physical fitness enhancer in different populations. Still, such studies generally require incorporating strict inclusion criteria to reduce potential bias when answering specific research questions [22, 23]. For example, meta-analyses would (understandably) exclude uncontrolled studies [16, 17, 24] and target specific outcomes (e.g., reactive strength index) [25] or populations (e.g., water sports athletes) [24]. However, many professional sports teams are reluctant to provide a control group for intervention-based studies, precluding the inclusion of studies with high-level athletes in these meta-analyses. Therefore, applying rigorous inclusion criteria may overlook pragmatic studies performed in elite sports. Further, detecting methodological limitations and gaps in the literature offers meaningful potential for advancements in the study of CCT and physical fitness adaptations. For example, even though several review articles are available in the literature [5, 7, 12, 16–21], many research questions surrounding the effectiveness of CCT still need to be addressed. For example, does the mode of muscle action (i.e., eccentric, isometric, concentric) moderate CCT effects? Can CCT benefit from resisted or assisted jumps at high or low loads? In this regard, a systematic scoping review is well-suited to detect methodological limitations and gaps in the literature and provide directions for future research.

Therefore, this systematic scoping review aimed to (a) characterize the main methodological features of the body of literature examining CCT (e.g., participant characteristics, training protocols, reporting of PAPE during training sessions), (b) map the existing evidence and identify relevant gaps (using an evidence gap map) in the literature, and (c) recommend future directions for CCT research. The information gathered from this systematic scoping review can support practitioners and sports scientists by consolidating information on this topic area while helping to address better the identified methodological limitations in future research.

## Methods

A systematic scoping review with an evidence gap map was conducted using the PRISMA 2020 [26] and PRISMA extension for Scoping Reviews [27] guidelines.

### Protocol and Registration

The protocol for this systematic scoping review was published in the Open Science platform (OSF) on March 2023 with the registration DOI number 10.17605/OSF.IO/BVRH8, archived link https://archive.org/details/osf-registrations-bvrh8-v1 and project link https://osf.io/xpw36/.

### Eligibility Criteria

Eligibility for inclusion in this scoping review was based on the PICOS (participants, intervention, comparator, outcomes, study design) approach (Table 1). The included cohorts eligible for inclusion were not restricted by age, sex, sports, or physical fitness criteria. Studies were included with interventions lasting four weeks or longer and if they incorporated contrast pairs of exercises (e.g., 80% 1RM squat combined with CMJs) as recommended by Cormier et al. [5]. The comparator groups were also not restricted for this scoping review. Hence, all studies that incorporated CCT were included. This allowed us to map the evidence level of the CCT studies (e.g., randomized, non-randomized with comparators, non-randomized without comparators). Regarding outcomes, studies that incorporated pre- and post-intervention testing in each outcome related (although not limited) to health-related fitness (e.g., muscular endurance, body composition), performance/skill-related fitness (e.g., power, speed), biomechanics (e.g., knee angle at landing, muscle activity), and physiology (e.g., oxygen uptake) were included. According to the principles of scoping reviews [28–30], involving those in the field of resistance training and plyometric jump training [1, 22], various study designs were considered for inclusion to produce a comprehensive evidence gap map. Furthermore, both non-randomized and randomized studies were included and are tagged accordingly in the evidence gap map. No restrictions as to language were implemented.

**Table 1:** Selection criteria used in the systematic scoping review

Category	Inclusion Criteria	Exclusion Criteria
**Population**	Any human population.	Non-human studies (e.g., rats).
**Intervention**	A complex-contrast training program is defined as a combination of heavy-load strength exercise alternated with low-load exercise, set by set (Thapa et al. 2021). Studies with at least four weeks of intervention were included.	Exercise interventions not involving complex-contrast training or exercise interventions involving other forms of complex training. E.g., descending training, where strength training exercises were conducted first, and ballistic exercises were performed at the end or during a different session.
**Comparator**	No restrictions (control [active, passive] and non-control studies).	—
**Outcome**	A priori, no limitation was considered for outcome inclusion, considering (but not limited to) physical fitness, health, psychological, and biomechanical outcomes.	Studies examining the acute effects of post-activation performance enhancement. Studies presenting only post-intervention values.
**Study design**	No restrictions.	—

### Search Strategy

#### Information Sources

A search was conducted in PubMed, SCOPUS, and Web of Science (core collection) electronic databases. A preliminary search was conducted on 10th March 2023, and updated on 20th February 2024. The studies published from the inception of each respective database until 20th February 2024 were considered for inclusion.

#### Search Process

The keywords were collected through expert opinions and previous systematic review studies on CCT [12, 16–19] and controlled vocabulary (e.g., Medical Subject Headings: MeSH). The search strategy for specific databases is provided in Supplementary File S1. In addition to database searches, manual checking of the reference lists of previous systematic review studies (identified during search strings) was performed. To further reduce the risk of publication bias, two renowned researchers in the field of CCT (identified through Scopus Researcher Discovery [www.scopus.com]) were contacted. They were asked to review the list of the 68 included studies (in line with the inclusion criteria) and to suggest additional studies (if applicable). The experts in the field suggested no additional studies. Finally, errata or retractions for the included studies were searched and considered (if applicable). In the case of retraction, the study was excluded from the review.

### Selection of Sources of Evidence

After an initial search, accounts were created in the respective databases. Through these accounts, an investigator (RRC) received automatically generated emails for updates regarding the search items used. Updates received beyond 20th February 2024 will be considered for future scoping review updates, which will be conducted when sufficient studies are available. After the search, two authors (RKT and RRC) screened the provisionally included studies based on the eligibility criteria using a two-stage approach. The first stage involved screening of articles based on titles and abstracts. The second stage involved the full-text analysis. Any discrepancies between the two authors were resolved through consensus with a third author (AW).

### Data Charting Process

Being a systematic scoping review [27, 31], ‘data’ refers to study characteristics and the dependent variables assessed in the study. However, the actual measurements (e.g., mean, standard deviation) derived from specific tests (e.g., 30 m linear sprint) were not extracted. All data (i.e., study characteristics and dependent variables) were coded into a specifically designed Microsoft^®^ Excel worksheet.

### Data Items Extraction

From potentially relevant retrieved studies, generic information (e.g., author name, journal name, year) and abstracts were saved for analysis using EndNote software (Clarivate, USA). Two authors (RKT and AW) independently processed all data, with one extracting and the other verifying. Several data items were considered for extraction based on previous recommendations for improving searching in electronic databases [32] and expert opinions on methodological gaps and limitations of CCT studies. Furthermore, the items were grouped into four broad subjects for results presentation and discussion purposes: (a) participants-related characteristics; (b) intervention-related information (key elements of CCT); (c) comparators (when available); and (d) outcomes. A detailed outline of these items is provided in Supplementary File S2.

### Synthesis of Results

A narrative synthesis was performed, accompanied by basic descriptive numerical summaries (e.g., number, percentage) for the previously defined data items to provide an overview of the existing body and the corresponding gaps in research. Interactive evidence gap maps were constructed using EPPI mapper software (Version 2.2.4). The gap maps graphically represent an overview of the existing evidence and current research gaps [33–35]. Firstly, the studies were coded in the EPPI-Reviewer 4 software (available at https://eppi.ioe.ac.uk/cms/Default.aspx?tabid=2914) in a customized code specifically prepared to elaborate an evidence gap map. A coding report (JSON) was created using the same software, which was then imported to EPPI mapper software to create an evidence gap map. Additionally, the level of evidence (i.e., higher for randomized versus non-randomized studies) was considered in the evidence gap maps (Supplementary File S7).

## Results

### Eligible Articles

**Fig. 1:**
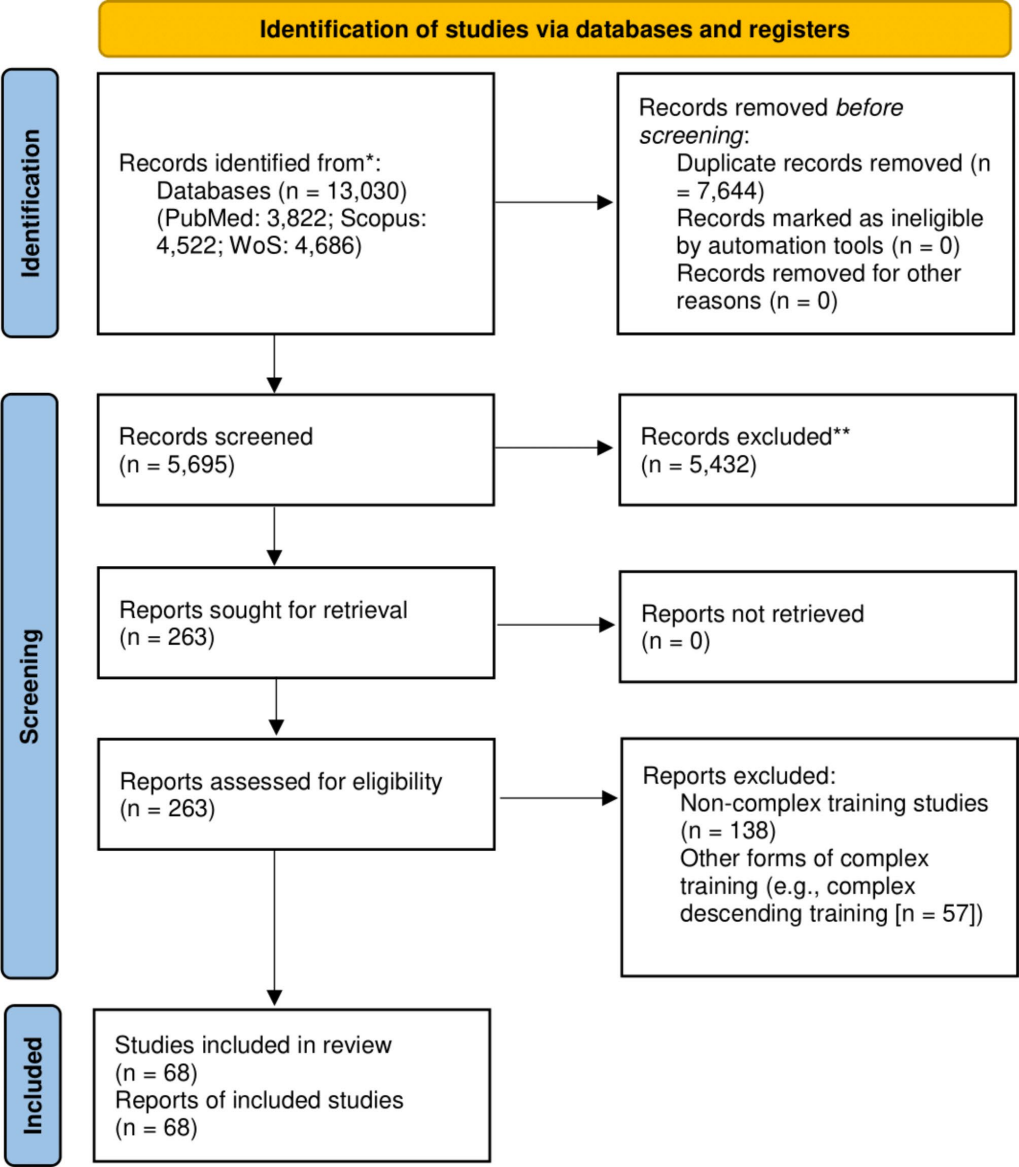
Flow chart illustrating the different phases of the search and study selection

From the 5,695 documents screened for inclusion, 263 articles remained after exclusion based on the titles or abstracts. After the full-text assessment of the remaining 263 articles, the list of the articles considered to be included was sent to two independent experts in the field of CCT, and these provided no further includable articles. Therefore, 68 articles were finally included in the scoping review. The flowchart of the screening process is represented in Fig. 1.

**Fig. 2:**
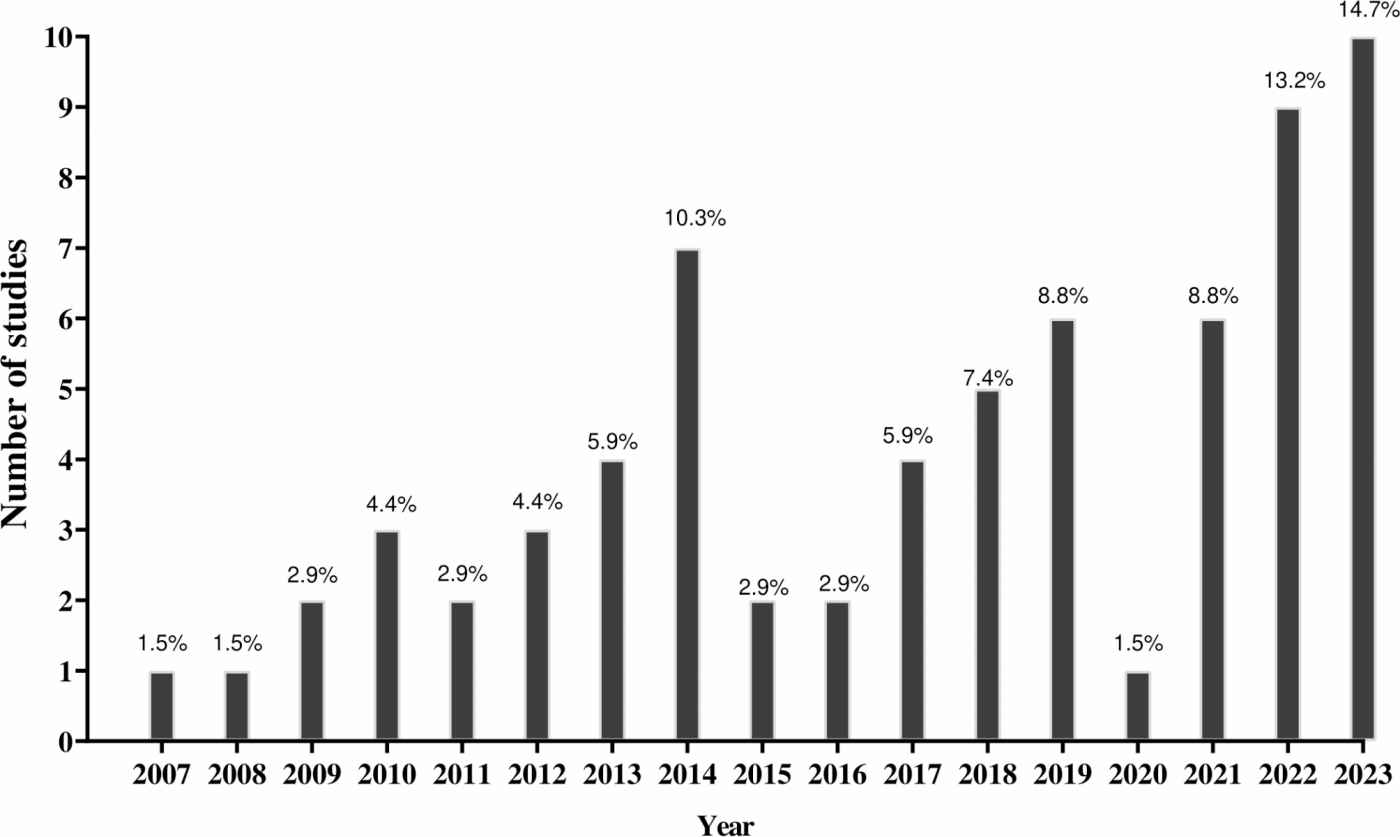
Number of studies published per year on complex contrast training

The included studies were published in 31 journals (Supplementary File S3). A graphical representation of the number of studies published per year is shown in Fig. 2.

### Participants Related Characteristics

A total of 77 experimental groups (CCT), 36 active control groups, and 35 specific-active control groups (e.g., traditional resistance training) were included in this study. The number of participants included per study averaged 11.3 (standard deviation = 4.6, median = 10, interquartile range = 6, range = 27), totaling 1,821 participants. Studies (79.4%) were conducted using male participants, 7.35% with female participants, and 10.3% including mixed participants (i.e., both males and females). Of note, 2.9% of the studies (2 of 68) did not clearly report participants’ sex. In total, 73.5% of the studies included participants aged *≥* 18, and 26.7% (18 studies) included participants under < 18. Only 6 [36–41] out of 17 studies reported participants’ maturation level. No study included a mixture of young (< 18 years) and adult (≥ 18 years) participants. According to the classification described by McKay et al. [42], 6 studies involved ‘tier 4’ (elite/international-level athletes), 25 studies involved ‘tier 3’ (highly-trained/national-level), 22 studies involved ‘tier 2’ (trained/developmental individuals), 14 studies involved ‘tier 1’ (recreationally active), and one study involved ‘tier 0’ (sedentary) level participants. No studies reported the inclusion of world-class athletes.

Among the included studies, participants were athletes who trained soccer (27.9% of the studies), rugby (10.29%), basketball (8.8%), volleyball (5.9%), endurance running (5.9%), and handball (4.4%). Furthermore, 1.5% of the included studies included participants involved in baseball, Australian rules football, fencing, gymnastics, pentathlon, or track and field (i.e., throwers). Moreover, 14.7% of the studies included physical education students, 4.4% included non-athlete recreationally active participants, and 1.5% included non-athlete sedentary participants. The screening of studies revealed that the participating individuals were experienced in resistance training in 55.8% of the included studies. Six studies reported ≥ 6 months, 14 studies ≥ 1 year, 5 studies ≥ 2 years, 2 studies ≥ 3 years, and 1 study reported ≥ 8 years of resistance training experience. 22% of the studies reported no resistance training experience, and another 22.0% provided no clear information regarding participant’s resistance training experience. More than half of the studies (70.6%) did not clearly report the previous training experience of the participants with the lower-load activity used in the study (e.g., plyometric training status). Only 13.2% of studies reported participants having previous ballistic training (e.g., plyometric training) experience, while 16.2% reported no prior experience. Furthermore, 85.3% of studies did not report the relative strength level of the participants. Among the 15.3% of the studies that measured participant’s relative strength, one research group reported back squat relative strength above ≥ 2.0 [43], three reported values of ~ 1.8 [44–46], and six studies reported values of ~ 1.5 [39, 47–51].

### Key Elements of Complex-Contrast Training Prescription Variables

The CCT intervention of the included studies was generally well-described (i.e., treatment description allows for adequate study replication, including duration, frequency, intensity, exercises, sets, and repetitions) (92.7%), with only 7.4% of the studies considered to have insufficiently described interventions (i.e., did not include any one of the five variables mentioned above). Notably, only two studies [52, 53] recorded PAPE before and after the training intervention, and none of the included studies recorded PAPE responses during the training session. More than half of the studies (70.6%) used lower-body exercises, while 29.4% included a mixture of upper and lower-body exercises. No studies specifically focused on upper-body exercises. Heavy-load exercise (e.g., ≥ 85% 1RM squat) was reported in 88.2% of the studies, while 1.5% used lower loads (e.g., 15–50% body mass) [54], optimal eccentric load (rate of force development at 0–200 ms during squats) [44] or accentuated eccentric load (105/90% 1RM) [49]. Heavy load isometric exercise was used by 4.4% of the studies [40, 45, 55], with 2.9% of the investigations using resistance bands (250% elongation) [36, 37] as the heavy-load exercise. Regarding lower-load exercises, body mass was used in 77.9% of the studies, while 19.1% used weighted exercises (e.g., squat jumps with 30% 1RM), and only 1.5% used assisted exercises (e.g., elastic bands).

One pair of CCT exercises (e.g., heavy squat combined with CMJ) was reported in 36.8% studies, 8.8% reported two, 26.5% reported three, 26.5% reported four, and 1.5% reported five pairs. The number of sets per CCT exercise at the start of interventions varied from 1 set (1.5% studies), 2 sets (10.3% studies), 3 sets (63.2% studies), 4 sets (13.2% studies), 5 sets (3.0% studies), 6 sets (5.9% studies), 7 sets (1.5% studies), up to 8 sets (1.5% studies). Progressive volume-based overload (number of sets) was reported in a small fraction of studies. Studies progressed from 2 to 3 sets (1 study), from 3 to 4–5 sets (9 studies), from 4 to 5–6 sets (4 studies), from 5 to 13 sets (1 study), and from 6 to 8 sets (3 studies)Regarding the number of repetitions used during CCT sets, 20.6% of studies used 6 repetitions, 11.8% of studies used 3 repetitions, 5.9% of studies used 4–5 repetitions, 2.9% studies used 2 repetitions, and 1.5% of studies used 7–12 repetitions. Other 45% of studies used different repetition ranges across the intervention, usually with a higher number of repetitions early during the intervention, reducing the number of repetitions toward the end of the intervention. Three studies reported isometric (high-load) exercises, with sets lasting between 3 up to 80 s.

#### Intra-Contrast Rest Interval

The intra-contrast rest interval (i.e., recovery period between the high-load and low-load exercises) was only reported in 57.4% of the studies. Five studies used no intra-contrast rest period (i.e., alternate exercise was performed immediately), while 29 studies used intra-contrast rest periods ranging from 30 to 480 s: 4 studies used 30 s, 5 studies used 60 s, 4 studies used 90 s, 2 studies used 120 s, 1 study used 150 s, 7 studies used 180 s, 4 studies used 240 s, 1 study used 300 s, and 1 study used 480 s. Two studies [50, 56] used an individualized intra-contrast approach (i.e., based on the PAPE duration). The rest between consecutive contrast sets was reported by 79.4% of the studies. Furthermore, the rest between consecutive contrast pairs (i.e., between two contrast exercises) was only reported in 23.5% of the studies. In 7.4% of the studies, the rest between contrast pairs was not applicable (e.g., only one contrast pair was prescribed).

#### Training Frequency and Duration

More than half of the studies incorporated two weekly sessions (73.5%), while 20.6%, 2.9%, and 1.5% incorporated three, one, or mixed two to three weekly sessions, respectively. Furthermore, interventions duration ranged from four to sixteen weeks, with more than half of the studies lasting ≤ 8 weeks, i.e. six weeks 36.8%, eight weeks 22.1%, four weeks 13.2%. Regarding the recovery between consecutive CCT sessions, 58.8% of the studies reported a minimum recovery duration of 48 h, followed by 72 h (10.3%) and 24 h (1.5%). Of note, 30.9% of the studies did not report the recovery hours between two consecutive CCT sessions.

#### Training Period and Intensity

Considering the studies involving athletes, 36.8% were conducted during the pre-season, 33.8% during the in-season, 5.9% during the off-season, and 13.2% did not report the training period. Regarding intensity, studies prescribed moderate-high intensity (55.9%) exercises, 19.1% high intensity, 16.2% moderate intensity, 5.9% low-moderate intensity, and only 1.5% low intensity. Furthermore, during the intervention, 66.2% of studies used a progressive overload approach in the form of intensity (13.2%), intensity and volume (39.7%), or volume alone (13.2%). Conversely, 33.8% of the studies used a non-progressive load approach in the intervention.

#### Training Surface

More than half of the studies did not clearly report the training surface for the lower-load, high-velocity activities (e.g., plyometrics) (98.5%). Only one investigation [56] reported the training surface (i.e., force platform) for the lower-load activity. The CCT was incorporated as a replacement for some part of training in 19.1% of the studies, while in 26.5%, CCT complemented the usual training program. Finally, tapering strategies (i.e., a reduction in training load) were included in only 8.8% of the studies.

### Comparators

A total of 78 experimental groups that performed CCT were included. An active control group was included in 50.0% of studies, while a specific-active control group was included in 64.7%. Two studies did not have any comparator group. The specific-active control groups included alternative forms of complex training (e.g., complex-descending training, complex-ascending training), traditional resistance training, compound training (i.e., resistance training and plyometric training performed on separate days), plyometric training, or different forms of CCT. A detailed list of evidence gaps regarding the comparator groups is provided in Supplementary Table S4.

### Outcomes

Twenty outcome variables were assessed across all the CCT studies. A detailed description of the prevalence of dependent variables assessed among the included studies is reported in Table 2. Briefly, CMJ performance was reported in 86.8% of the studies, linear sprint in 58.8% of the studies, dynamic strength in 51.5% of the studies, change of direction speed (CODS) in 45.6% of the studies.

**Table 2:** Number of studies that assessed selected physical fitness parameters

Variable	Studies (*N* = 68) (%)
Countermovement jump	59 (86.8)
Sprints	40 (58.8)
Dynamic strength	35 (51.5)
Change of direction speed	31 (45.6)
Squat jumps	27 (39.5)
Horizontal jumps (e.g., standing long jump)	15 (22.1)
Dynamic strength upper-body	14 (20.6)
Drop jumps	11 (16.2)
Isometric strength	11 (16.2)
Muscle properties^a^ (e.g., muscle thickness)	11 (16.2)
Muscle strength in isokinetic mode	9 (13.2)
Body fat percentage	8 (11.8)
Aerobic endurance	8 (11.8)
Sport-specific performance (e.g., kicking speed in soccer)	7 (10.3)
Repeated sprint ability	7 (10.3)
Biochemical (e.g., creatine kinase)	5 (7.4)
Balance	4 (5.9)
Electromyography	2 (2.9)
Leg stiffness	1 (1.5)
Interlimb asymmetry	1 (1.5)

^a^: includes (but is not limited to) muscle hypertrophy, pennation angle, muscle thickness, and cross-sectional area.

### Evidence Gap Maps

Three interactive evidence gap maps were created regarding participants’ sex (Supplementary File S5), participants’ age (i.e., < 18 vs. ≥18 years) (Supplementary File S6), and study design (e.g., randomized controlled or non-randomized controlled) (Supplementary File S7).

## Discussion

This systematic scoping review aimed to (a) characterize the main methodological features of the literature available on CCT, (b) map the existing evidence and identify relevant gaps in the literature, and (c) provide recommendations for future CCT studies. The main methodological features of the literature available on CCT are discussed in detail (as subsections), with mapping of existing evidence for each methodological feature, identification of relevant gaps, and recommendations for future research wherever possible. One of the main findings was limited number of studies being conducted in females as well as in youths. Additionally, although PAPE is commonly suggested to be one of the mechanisms mediating the adaptations observed after CCT interventions, another key finding was the absence of PAPE measurements during the CCT sessions or pre-post-training interventions. Furthermore, intra-contrast rest intervals (i.e., recovery between the heavy-load and lower-load exercise) were not clearly reported in ~ 41% of studies. The CCT effects were compared with other types of complex training methods in 38% of the studies. Outcome variables reported in ≥ 50% of the studies were CMJ, sprint performance, and dynamic strength. Furthermore, there is a lack of long-term studies (e.g., > 16 weeks) implementing CCT. Following this general appraisal, a thorough discussion regarding evidence gaps and future research directions is provided, focusing on CCT programming (prescription) variables such as duration, intensity, frequency, and comparators.

### Training Duration

The CCT interventions lasted four – five weeks (*n* = 11), six weeks (*n* = 25), seven – eight weeks (*n* = 18), nine – ten weeks (*n* = 8), and twelve – sixteen weeks (*n* = 6). The analysis of outcomes according to the duration of the CCT in included studies are depicted as evidence gap maps for sex, age, and study design in Supplementary files S5, S6, and S7, respectively.

Although the minimal effective intervention dose is multifactorial (e.g., training intensity; training volume), training duration (number of weeks) is a particularly relevant factor. For example, jump performance can improve after two weeks of training [57], while bone density may require a considerably longer adaptation time (e.g., 3 to 4 months for one remodeling cycle to complete the sequence of bone resorption, formation, and mineralization) [58]. In clinical contexts, patients may seek a fast discharge from the clinical setting. Similarly, injured athletes under rehabilitation need to know the time (weeks) required to return to sport. Further, athletes aiming for key competitions (e.g., Olympics) may benefit from a temporal periodization of CCT protocols to manipulate the training stimulus within the macro- or mesocycle. Therefore, the minimal effective CCT duration reported in the included studies was retrieved. From the 20 outcomes included in our review, 13 were assessed after CCT interventions lasting four weeks. Improvements were reported in CMJ [9, 11, 47, 50, 56, 59–64], SJ [47, 60], SLJ [11, 59, 63, 64], linear sprints [11, 50, 59, 60, 63, 64], lower-body dynamic strength [47, 50, 60], upper-body dynamic strength [60], body fat percentage [50], and aerobic endurance (e.g., Yo-Yo intermittent recovery level 1) [60].

Longer-term studies (i.e., > 4 weeks) also reported improved physical fitness outcomes (e.g., SJ, CMJ, DJ) (Supplementary files S5-S7). The outcome variables that require the strength-power complex (e.g., jumps, sprints, 1RM strength) were improved across four – sixteen weeks. Furthermore, muscle properties such as triceps surae girth [65], type I, type IIA, and type IIX muscle fiber cross-sectional area [44], vastus lateralis thickness [66], and leg stiffness [67] were also improved after six-week CCT interventions in males with positive adaptations observed in the thigh muscle volume after eight- [68], ten- [41], and sixteen-week CCT [69] programs. In contrast, no improvements were observed in muscle mass [70, 71] and type I, type IIA and type IIX muscle fiber percentage [44] after six weeks of CCT. Remarkably, no evidence for muscle properties was available among female participants across any training duration.

Reduced fat percentage was observed in adult males after four weeks of CCT in combination with creatine supplementation during a diet-controlled approach [50]. Conversely, in non-diet controlled studies, no changes in the fat percentage were observed following five- [62], six- [65, 70–72], eight- [73], or sixteen-week [69] CCT interventions, including males (1 youth study, 5 adult studies) or mixed-sex (adult) participants. Furthermore, one study [74] reported improved shooting efficiency in soccer after six-weeks of CCT, while three studies reported improvement in running economy (i.e., sport-specific skills) after CCT durations of six [72], eight [73], and twelve weeks [75]. Shot put throwing and soccer kicking ball velocity were improved following eight [8] and twelve weeks [55] of CCT. Furthermore, an increase in free testosterone was observed in adult male participants (age range 18–24 years) when a six-week CCT intervention was prescribed [48].

Of interest, when considering outcomes assessed in ≥ 2 independent randomized-controlled studies, critical evidence gaps were noted. Firstly, no evidence was available for adult females. Secondly, youth females and youth males were only assessed in CCT interventions that lasted ten and eight weeks, respectively, and only for 9 and 7 (respectively) of the 20 outcomes included in the evidence gap maps. Thirdly, adult males were only assessed in CCT interventions that lasted 6–7 weeks and only for 3–5 of the 20 outcomes included in the evidence gap maps. Therefore, outcomes were reported after interventions that lasted between four to sixteen weeks, although reports usually involved only a single randomized-controlled intervention, precluding comparative-confirmative analyses. Further, none of the outcomes included in the evidence gap maps attained a sample size involving ≥ 800 participants [76], reducing the current evidence’s confidence. Therefore, although 20 outcome-related measures were reported across included studies in adults, youths, males, or females, after interventions of four – sixteen weeks, the robustness of current recommendations for a minimal effective CCT duration (or expected long-term [e.g., > 10 weeks] adaptations) to improve jumping, sprinting, strength, and related physical fitness traits, is currently low. Future high-quality (e.g., randomized-controlled) CCT studies involving larger samples (particularly females) and varied duration (particularly longer-term) are needed to fill the evidence gaps noticed in this systematic scoping review.

### Training Intensity

Three randomized controlled trials [67, 77, 78], three randomized non-controlled [49, 79, 80], one non-randomized non-controlled [81], and one study with unclear study design [82] compared different CCT intensities. The studies involved 17 experimental groups and 4 control groups, 222 male participants, and assessed 10 different outcomes. Three studies compared traditional CCT (e.g., 93% 1RM) with variable-resistance-based CCT (i.e., 70% 1RM, combined with an elastic resistance band equivalent to 0–23% 1RM) over six weeks [67, 78, 79]. Researchers from these studies reported similar improvements in back squat 1RM, linear sprint speed, CMJ height, and muscle properties (e.g., muscle thickness, pennation angle) [67, 78, 79]. Additionally, Argus et al. [80] compared four weeks of strength-power (80–95% 1RM) vs. speed-power (55–65% 1RM) box squats (as the high-load exercises of the CCT pair), with the former CCT intervention inducing greater improvements (of small magnitude) for CMJ, SJ, and horizontal jump performance. Further, Smith et al. [77] compared six weeks of squat vs. kettlebell swing (as the high-load exercises of the CCT pair), and reported similar improvements in vertical jump performance. Furthermore, Smilios et al. [82] reported similar improvements in lower-body strength and CMJ performance after three CCT interventions: SJs performed with a heavy load (90% 1RM) vs. external load (minus body mass) at maximum power vs. external load (plus body mass) at maximum power. In contrast, McMaster et al. [81], having 85% 1RM as the conditioning activity load (i.e., first exercise of contrast pair), compared the use of light (15–30% 1RM) vs. moderate-heavy (60 − 70% 1RM) loads on the second exercise of the contrast pair (e.g., SJs, bench press throws) and reported improvement in lower and upper-body strength, and reductions in sprint time in both groups. Lastly, Chakshuraksha et al. [49] compared six weeks of traditional (90/90% 1RM eccentric/concentric) vs. accentuated eccentric loading-based CCT (i.e., 105/90% of 1RM eccentric/concentric) and reported greater improvement in lower-body strength and CODS in the former loading condition. Based on the available evidence, CCT with traditional resistance exercise (i.e., ≥ 80% 1RM) or variable-resistance exercise (i.e., 70% 1RM, combined with elastic resistance band equivalent to 0–23% 1RM) seems effective as the heavy load activity (i.e., first CCT exercise). For the lower load activity (i.e., second CCT exercise), ballistic exercises (e.g., 0% body mass; 15–30% 1RM; 60–70% 1RM SJ) seems effective.

Still, there were critical evidence gaps noted concerning training intensity. For example, only three randomized controlled studies were available, leading to evidence gaps for each outcome variable. Secondly, no studies were conducted on female participants across age groups or young males (< 18 years). Thirdly, the total sample size reached only 250 participants for all included studies, further reducing the robustness of the evidence. Also, only one study compared the intensity of the second exercise of the contrast pair (i.e., low-load exercise). Fourthly, studies do not precisely describe training intensity using an objective measure for lower-load exercises. Lastly, one study incorporated a magnitude-based inference approach [80] that has been recently criticized [83]. Therefore, future high-quality studies (randomized controlled trials) should compare the effects of different intensities of both high-load (i.e., primary) and low-load (i.e., secondary) exercises across sex and age (particularly focused on youth and adult females and male youths) so that it is possible to obtain further knowledge on the effectiveness of CCT interventions.

### Training Frequency

Only three studies [51, 74, 84] compared CCT frequencies. Moreover, from the 20 outcomes analyzed in this scoping review, only 8 were analyzed regarding the effects of CCT frequency (Table 2). Further, the three studies included only male participants (2 young and 1 adult). Of the three studies, two were randomized controlled [51, 74], and one did not clearly report the study design [84]. Cavaco et al. [74] compared 1 vs. 2 CCT weekly sessions and reported similar improvements in sport-specific outcomes in both training frequencies. Similarly, Alves et al. [84] compared 1 vs. 2 CCT weekly sessions and reported similar improvement in linear sprint and SJ with both training frequencies. Nonetheless, in both studies, the authors did not report whether the total training volume was equated. Kumar et al. [51] compared 2 vs. 3 volume-equated weekly CCT sessions and reported superior improvements in sprint and CODS with the latter training frequency, while greater gains in CMJ and horizontal jump performances were observed in the former condition. Isokinetic strength, on the other hand, had similar improvement with both volume-equated training frequencies. Based on the available evidence, 2–3 weekly CCT sessions seem effective. However, in case of a congested training week, a single weekly CCT session may also be effective in improving physical performance.

Again, it is important to note that (a) there is a scarce number of studies comparing the effects of different CCT training frequencies on participants’ physical fitness [51, 74, 84]; (b) no study was found with female participants across age groups; (c) only 8 of the 20 assessed outcomes in our systematic scoping review were analyzed according to CCT frequencies; (d) of the three studies two did not report if volume was equated. For these reasons, within the current state of the literature, it is difficult to draw meaningful conclusions about whether training frequency is a key aspect to consider when programming CCT interventions. As such, future studies should investigate the physiological and performance-based adaptations following CCT programs with higher or lower weekly frequency and clearly report whether the volumes are equated.

### Comparators (specific-active)

#### CCT Versus Traditional Resistance Training

Seventeen studies compared CCT with traditional resistance training (14 male, 2 female, 1 mixed), with five randomized controlled [48, 60, 68, 75, 85], nine randomized non-controlled [43, 59, 65, 70, 73, 86–89], one non-randomized controlled [72], one non-randomized non-controlled [90], and one study not clearly reporting the randomization process [91]. The total number of outcome measures assessed was 16 out of 20. More than half of the studies that compared CCT with traditional resistance training across four to twelve weeks of duration reported non-significant differences between both the training methods for CMJ performance [43, 48, 60, 72, 89, 91], SJ height [60], horizontal jump distance [59], lower-body dynamic strength [43, 65, 70, 72, 89, 91], linear sprinting ability [43, 48, 59, 89, 91], muscle properties (e.g., thigh girth) [65], CODS [48, 59, 89, 91], sport-specific performance (e.g., running economy) [72], or aerobic endurance [60]. Still, comparisons between the within-group results may suggest greater magnitudes (ES) of improvements following CCT over traditional resistance training for CMJ (large vs. trivial [86]), SJ (large vs. trivial [87]), linear sprints (small vs. trivial [87]), CODS (large vs. trivial [87]), and aerobic endurance (moderate vs. small [60]). However, a comparison of within-group ES results should be interpreted with caution due to a high risk of misleading results [13]. Of note, one study compared eight weeks of CCT versus traditional resistance training using a within-subject design (with a two-week washout period), and reported greater improvements after CCT in CMJ, SJ, lower-body strength (i.e., 1RM squat, isometric mid-thigh pull), and the reactive strength index [90]. Based on the available evidence, both CCT and traditional resistance training seem to offer similar performance enhancements.

#### CCT Versus Plyometric Training

Seven studies (6 male and 1 female) with durations ranging from four to eight weeks compared CCT with plyometric training alone, which included three randomized-controlled [85, 92, 93], three randomized non-controlled [59, 65, 89], and one without a clear description of randomization process [56]. Of the seven studies, only one included youth male participants [92]. Studies reported no between-group difference in CMJ outcomes [56, 59, 89, 93], horizontal jump [59], linear sprints [59, 85, 89, 92], CODS [59, 92], lower-body dynamic strength [65, 85, 89], upper-body strength [93], muscle properties [65] and aerobic endurance [93]. However, significant positive improvements in CMJ, SJ, lower-body dynamic strength, and electromyographical activation of thigh muscles (e.g., vastus medialis, rectus femoris) were observed in CCT compared to plyometric training in young males after 8 weeks [92]. Based on the available evidence, both CCT and plyometric training seem to induce similar performance enhancements.

#### CCT Versus Compound Training

Four studies (1 male, 1 female, and 2 mixed) compared CCT with compound training (i.e., traditional resistance training and ballistic training performed on separate days) which included two randomized non-controlled [8, 89], one non-randomized non-controlled [9], and one study that did not clearly report the randomization process [66]. All studies were conducted on adult participants, with three studies involving trained athletes (Australian football, volleyball, track and field throwers) and one sedentary population. Overall, there were no significant differences between CCT and compound training in improving CMJ [9, 89], linear sprint [89], CODS [89], lower-body dynamic strength [66, 89], or muscle properties (e.g., vastus lateralis thickness) [66]. Of note, one study reported compound training to be significantly more effective in improving CMJ performance [66], while another study reported CCT to be significantly better in improving lower-body dynamic strength and sport-specific performance (e.g., javelin throw performance) [8]. The results found in the literature preclude a recommendation of one training method over another for the improvement of physical fitness. However, CCT could be considered more time-efficient given that both high- and low-load explosive exercises are completed in the same training session. Hence, in contexts where the time available to devote to strength and conditioning sessions is limited, CCT could be a more suitable alternative.

#### CCT Versus Complex-Descending or Ascending Training

Six studies (sex: 5 males, 1 mixed; age: 5 adult, 1 youth) consisting of four randomized non-controlled [53, 94–96], one non-randomized controlled [97], and one non-randomized non-controlled [98] design compared CCT with either complex-descending or complex-ascending training. The CMJ, SJ, CODS, linear sprint, lower-limb dynamic strength, and isokinetic strength gains were similar irrespective of the intra-session exercise order [53, 94, 95, 97]. The only exception was the study by Dobbs et al. [98] that used the magnitude-based inference approach and reported greater meaningful improvement with CCT for CMJ and horizontal jumps compared to complex descending training. However, the use of magnitude-based inference approach in sport science has been criticized [83]. Moreover, a previous meta-analysis [12] reported the CCT format to be superior compared to other complex training formats, however, the meta-analysis did not use a direct comparison (e.g., CCT vs. complex-descending) and used the magnitude-based approach to make this conclusion. Therefore, the literature is still unclear whether CCT may be superior to complex-descending or ascending formats; hence, more comparative studies are necessary for a robust conclusion.

#### Different Forms of CCT

Nineteen studies compared different forms of CCT (e.g., traditional vs. blood flow restriction). From these, eight [49, 61, 67, 77–82] compared CCT performed with different training intensities, while three studies [51, 74, 84] compared interventions with different frequencies. Therefore, these studies are discussed in Sect. 4.2 and 4.3 and are not analyzed here. From the remaining investigations, seven studies (sex: 5 males, 1 mixed, 1 not clearly reported; age: 2 youth, 5 adults) compared different forms of CCT, which included 3 randomized non-controlled [41, 50, 99], and 4 non-randomized non-controlled [45, 46, 69] studies.

Thapa & Kumar [46] compared the effect of six weeks of CCT on stronger (relative strength ≥ 1.75) versus weaker (relative strength < 1.55) individuals and reported no between-group difference in post-training CMJ, horizontal jump, and linear sprint performances, and lower-limb isokinetic strength. Argus et al. [61] compared CCT that included either assisted (i.e., elastic bands), free (i.e., bodyweight; without any assistance or resistance), or resisted jumps as the low-load exercise of the contrast pair and reported (using a magnitude-based approach) similar small improvements in the assisted and resisted jumps conditions, while trivial improvements were reported with free jumps. Wang et al. [50] compared the effects of CCT with creatine intake and reported a significant increase in lower-body dynamic strength with a decrease in creatine kinase (i.e., a blood marker of muscle damage) during the first and last training sessions compared to the CCT with placebo intake. However, no differences were observed for CMJ, linear sprint, and body fat percentage. Bogdanis et al. [45] compared low-volume CCT with isometric leg press at two different joint angles (85° ± 2° vs. 145° ± 2°) as the high-load, low-velocity exercise and reported similar improvements in CMJ and lower-body dynamic strength performance. However, the isometric strength improved specifically at knee flexion angles close to the ones used in each training intervention. Furthermore, Gonzalez-Rave et al. [69] compared the effects of CCT on master athletes and physically active older adults (i.e., non-athletes) and reported significant improvement in CMJ, SJ, drop jump (DJ) performance, and muscle properties (e.g., muscle mass, thigh cross-sectional area) in both groups. Nonetheless, greater percentage changes were observed in CMJ (21.5% vs. 14.8%) and DJ (26.5% vs. 7.4%) in non-athletes compared to athletes. Furthermore, biochemical outcome variables such as creatinine (8.65%) and creatine kinase (25.49%) were significantly reduced only in athletes. Lastly, Gee et al. [99] compared the effects of a “traditional” CCT vs. a reverse-CCT (i.e., the low-load exercise was performed first, followed by high-load exercise in a set-by-set fashion) and reported no between-group difference in physical fitness assessed via vertical jumps, linear sprints, CODS, and upper-body strength (i.e., medicine ball throw).

A closer look at the current state of the literature highlights evidence gaps at each comparator level (e.g., traditional resistance training, plyometrics), as more than half of the available studies consists of non-controlled or non-randomized studies. For example, out of the 17 studies comparing CCT with traditional resistance training, 10 were from non-controlled or non-randomized studies. Similarly, the majority of the studies that compared CCT with plyometric training (3 out of 6), compound training (3 out of 4), complex ascending or descending training (4 out of 4), and other forms of CCT (6 out of 6), respectively, were either non-randomized or non-controlled. Therefore, future studies should consider using randomized controlled designs for a more robust evidence base. Furthermore, the low number of participants in the studies available that compared CCT with plyometrics (*n* = 217), compound (*n* = 105), or complex-ascending or descending training (*n* = 101) may conceivably contribute to the similar improvement reported across comparators. For this reason, more high-quality studies (leading to greater sample sizes) must be conducted with each comparator. Finally, the need for studies on female and youth participants should also be considered when future studies are designed.

### Other Evidence Gaps and Future Directions for Research

After analyzing the content of all CCT studies, other evidence gaps must be addressed. Firstly, the use of CCT needs to be examined across various phases of a periodized training plan (e.g., pre-season period, competitive period). Secondly, no study investigated the effects of incorporating different intra-contrast rest intervals (e.g., 30 s vs. 4 min) as part of a several-week CCT program. Thirdly, no research compared tapering vs. non-tapering approaches. Also, no study measured the PAPE effects during CCT sessions. As previous studies have consistently used PAPE as the mechanistic rationale for improvements observed following CCT interventions, it is of utmost importance that future research includes the assessment of PAPE during CCT sessions to verify this hypothesis. Additionally, as PAPE is mediated by the strength level of the participants [100, 101], future CCT studies should report the strength of the participants. Furthermore, although 20 studies used a mixture of lower- and upper-body contrast pairs (more than half of these included one upper-body contrast pair), no previous research investigated upper-body CCT interventions alone. Upper-body CCT may be a useful strategy for sports with a predominant reliance on upper-body power (e.g., rowing). Furthermore, future studies should clearly define the type of exercises (e.g., upper-body, lower-body, or combined) used and should precisely control the training loads with objective measures. Lastly, the number of participants did not reach the minimum threshold of 800 [76] in any of the assessed outcome variables for the discussed programming aspects (i.e., duration, intensity, frequency, and comparators), further limiting the generalization of the current evidence. Therefore, future high-quality randomized controlled studies should be conducted across different training durations, intensity, frequencies (volume-equated), and comparators with more outcome variables being measured.

## Conclusions

This systematic scoping review compiled the studies available for CCT across different databases from inception till 20th February 2024. A total of 68 studies were included, which comprised participants from different age groups, sex, sports, and expertise levels, with CCT durations ranging between four to sixteen weeks. Overall, the current confidence and generalization of the results based on the available evidence on CCT for each assessed outcome variable is still low due to the limited number of participants involved (< 800). Therefore, more studies are required in each of the discussed programming variables (e.g., duration, intensity, frequency) to enhance the level of evidence. Moreover, it is also important to note that no study assessed the PAPE during training sessions, making it difficult, with the current state of the literature, to attribute the improvements observed after CCT to PAPE. Lastly, we also provided interactive evidence gap maps as supplementary material S5 – S7 to give an overview of the evidence gaps in literature across age, sex, and study design.

## Electronic Supplementary Material

Below is the link to the electronic supplementary material.

Supplementary Material 1

Supplementary Material 2

Supplementary Material 3

Supplementary Material 4

Supplementary Material 5

Supplementary Material 6

Supplementary Material 7

## Data Availability

All data generated or analyzed during this study are included in the article as Table(s), Figure(s), and/or Electronic Supplementary Material(s). Any other data requirement can be directed to the corresponding author upon reasonable request.

## Footnotes

1 Weaker individuals targeting maximal strength development may also improve rate of force development and power production capacity.

2 The force-velocity curve is the inverse relationship between force and velocity. The force-velocity relation denotes reduced movement velocity at high forces and reduced force output at high movement velocities.

## Abbreviations

1RM: One repetition maximum
CCT: Complex–contrast training
CMJ: Countermovement jump
CODS: Change of direction speed
DJ: Drop jump
ES: Effect size
OSF: Open science framework platform
PAPE: Post activation performance enhancement
PICOS: Participants, intervention, comparators, outcomes, and study design
SJ: Squat jump
