# Supplementary material for: What do we Know about Complex-Contrast Training? A Systematic Scoping Review
Source: Sports Med Open. 2024 Sep 27;10:104. doi: 10.1186/s40798-024-00771-z (PMC11436572; doi:10.1186/s40798-024-00771-z)
Supplement: Supplementary file 1 — Supplementary Material 1 [file 40798_2024_771_MOESM1_ESM.docx]

| **Supplementary Table S1.** Search strategy used for different databases. | | | |
| --- | --- | --- | --- |
| **Search Date** | August 2021 | August 2021 (updates recorded until March 2023) | August 2021 (updates recorded until March 2023) |
| **Databases** | **PubMed** | **WoS (Core Collection)** | **Scopus** |
| **Keywords** | “complex training”, “contrast training”, “power”, “plyometric”, “resistance exercise”, “resistance training”, “resisted training”, “French contrast” | | |
| **Database fields for the search** | Title, abstract | All | Title, abstract, keywords |
| **Restrictions for the search** | None | None | None |
| **Example of search strategy code line** | "plyometric exercise"[MeSH Terms] OR ("plyometric"[All Fields] AND "exercise"[All Fields]) OR "plyometric exercise"[All Fields] OR ("plyometric"[All Fields] AND "training"[All Fields]) OR "plyometric training"[All Fields] | (ALL=(plyometric)) AND ALL=(training) | TITLE-ABS-KEY (plyometric AND training) |

**Electronic Supplementary Material Table S1**

**Article title**: What do we know about complex-contrast training? A systematic scoping review

**Author names**: Rohit K. Thapa, Anthony Weldon, Tomás T. Freitas, Daniel A. Boullosa, José Afonso, Urs Granacher, Rodrigo Ramirez-Campillo

**Affiliation and e-mail of the corresponding author**:

Prof. Urs Granacher, PhD

Department of Sport and Sport Science,

Exercise and Human Movement Science,

University of Freiburg, Freiburg, Germany.

[urs.granacher@sport.uni-freiburg.de](mailto:urs.granacher@sport.uni-freiburg.de)
