# Supplementary material for: What do we Know about Complex-Contrast Training? A Systematic Scoping Review
Source: Sports Med Open. 2024 Sep 27;10:104. doi: 10.1186/s40798-024-00771-z (PMC11436572; doi:10.1186/s40798-024-00771-z)
Supplement: Supplementary file 2 — Supplementary Material 2 [file 40798_2024_771_MOESM2_ESM.docx]

| **Supplementary Table S2.** Items* considered for data extraction from eligible studies. | | | |
| --- | --- | --- | --- |
| **Participant information** | **Intervention-related information (key elements of complex-contrast training)** | **Comparators** | **Outcomes** |
| (1) *Sex.* ‘male’ or ‘female’ or ‘mixed’.  (2) *Chronological* *age.*  (3) *Body mass.*  (4) *Body* *height.*  (5) *Playing level.* Participants were classified according to the following tier classification [1] (a) world-class; (b) elite/international level; (c) highly training/national level; (d) trained/developmental; (e) recreationally active; (f) sedentary.  (6) *Sports practiced.* If participants practiced a given sport, this was registered. If participants did not practice competitive sports, although they were physically active (e.g., recreationally active, physical education students, firefighters), or were identified as sedentary or physically inactive, this was also registered.  (7) *Systematic strength training experience before intervention.* If participants had previous experience, a dichotomous characterization identifier was used as ‘yes’ or ‘no’ without consideration of the extent of the experience.  (8) *Systematic ballistic training experience before intervention.* If participants had previous experience, a dichotomous characterization identifier was used as ‘yes’ or ‘no’ without consideration of the extent of the experience.  (9) *Training period of the season.* Where competitive athletes were recruited, the training period was identified as pre-season, in-season, and off-season. If participants were not in a competitive schedule (e.g., physical education students), the training period was recorded as ‘not applicable.’ | (1) *Training duration.*  (2) *Frequency.*  (3) *Intensity*. For heavy-load exercise, the intensity was recorded as (a) heavy (≥80% 1RM or ≤8 repetitions maximum); (b) moderate (60 – 79% 1RM or 9 – 15 repetitions maximum); (c) light (<60% 1RM or >15 repetitions maximum) [2]. For the low-load ballistic exercise, the intensity was recorded as (a) low; (b) moderate; (c) low to moderate; (d) high; (e) moderate to high; (f) very high [3].  (4) *Progressive overload.* Where the application of load progression was administered, categorized as ‘volume-based’ or ‘intensity-based.’ When training loads were constant across time, it was reported as ‘non-progressive’.  (5) *Exercise* *prescription*. The number of CCT exercises per session was recorded. Where possible, the type of CCT-exercise was recorded (e.g., squats with countermovement jump). The number of sets and repetitions per set were also recorded for each CCT-exercise.  (6) *Taper.* Where the application of a taper was administered, identified through a reduction in the training load in the latter parts of the training program (most often the last week of training). The duration and type (e.g., volume and intensity) of taper were registered when reported.  (7) *Rest between intra-contrast set and exercise, and training sessions.* The rest time between two intra-contrast sets (seconds), exercises (seconds), and training sessions (hours) was recorded.  (8) *Intra-contrast rest.* The rest period (seconds) between the two contrast exercises was recorded.  (9) PAPE during a training session | When available, comparators were recorded.  (1) Other complex training modalities (e.g., complex-ascending training, complex descending training, or French contrast training)  (2) Other strength training modalities (e.g., heavy resistance training, plyometric jump training)  (3) Different forms of exercises used for the heavy-load exercise within complex-contrast training. | All the outcomes reported in the studies were extracted.  (1) Physiological (e.g., muscle fiber diameter).  (2) Performance-related (e.g., CMJ height; CMJ force; range of motion).  (3) System level(s) (e.g., cardiovascular, musculoskeletal, nervous).  (4) Psychological (*e.g.,* perceived well-being, RPE).  The performance-related outcomes were also analyzed according to their factor emphasis (e.g., strength, flexibility) to provide an overview of which categories were assessed. Considering the goal of providing a systematic scoping review with an evidence-gap map, outcomes were registered, but results were not. For example, registering that *k* studies assessed the CMJ, but the actual measurement values were irrelevant for our purposes. |
| 1RM – one repetition maximum, CCT – complex-contrast training, CMJ – countermovement jump, PAPE – post-activation performance enhancement, RPE – rating of perceived exertion  *Items considered for data extraction from eligible studies when not clearly reported were registered as “not reported”. | | | |

**References**

1. McKay AKA, Stellingwerff T, Smith ES, Martin DT, Mujika I, Goosey-Tolfrey VL, et al. Defining Training and Performance Caliber: A Participant Classification Framework. Int J Sports Physiol Perform. 2022 Feb 1;17(2):317-31.

2. Lopez P, Radaelli R, Taaffe DR, Newton RU, Galvão DA, Trajano GS, et al. Resistance Training Load Effects on Muscle Hypertrophy and Strength Gain: Systematic Review and Network Meta-analysis. Med Sci Sports Exerc. 2021 Jun 1;53(6):1206-16.

3. Ramirez-Campillo R, Álvarez C, García-Hermoso A, Ramírez-Vélez R, Gentil P, Asadi A, et al. Methodological Characteristics and Future Directions for Plyometric Jump Training Research: A Scoping Review. Sports Med. 2018 May;48(5):1059-81.
