# Supplementary material for: What do we Know about Complex-Contrast Training? A Systematic Scoping Review
Source: Sports Med Open. 2024 Sep 27;10:104. doi: 10.1186/s40798-024-00771-z (PMC11436572; doi:10.1186/s40798-024-00771-z)
Supplement: Supplementary file 3 — Supplementary Material 3 [file 40798_2024_771_MOESM3_ESM.docx]

| **Supplementary Table S3.** Journals articles were published in | |
| --- | --- |
| **Journal Name** | **No** |
| Journal of Strength and Conditioning Research | 19 |
| International Journal of Sports Physiology and Performance | 7 |
| Journal of Human Kinetics | 3 |
| Journal of Sports Medicine and Physical Fitness | 3 |
| Journal of Sports Sciences | 3 |
| European Journal of Sport Science | 2 |
| Frontiers in Physiology | 2 |
| International Journal of Sports Science and Coaching | 2 |
| Isokinetic and Exercise Science | 2 |
| Journal of Australian Strength and Conditioning | 2 |
| Sports | 2 |
| Theory and Methods of Physical Education | 2 |
| Biomedical Human Kinetics | 1 |
| BMC Sports Science, Medicine and Rehabilitation | 1 |
| Facta Universitatis | 1 |
| Frontiers in Psychology | 1 |
| Human Movement | 1 |
| International Journal of Environmental Research and Public Health | 1 |
| Journal of Chiropractic Medicine | 1 |
| Journal of Exercise Physiology Online | 1 |
| Journal of Musculoskeletal Research | 1 |
| Journal of Science in Sport and Exercise | 1 |
| Montenegrin Journal of Sports Science and Medicine | 1 |
| Medicine and Science in Sports and Exercise | 1 |
| Nutrients | 1 |
| PeerJ | 1 |
| Polish Journal of Sports Tourism | 1 |
| Scandinavian Journal of Medicine & Science in Sports | 1 |
| Sport Sciences for Health | 1 |
| Sportverl Sportschad | 1 |
| Studia Sportiva | 1 |
