# Supplementary material for: What do we Know about Complex-Contrast Training? A Systematic Scoping Review
Source: Sports Med Open. 2024 Sep 27;10:104. doi: 10.1186/s40798-024-00771-z (PMC11436572; doi:10.1186/s40798-024-00771-z)
Supplement: Supplementary file 4 — Supplementary Material 4 [file 40798_2024_771_MOESM4_ESM.docx]

| **Supplementary Table S4.** Evidence gaps regarding the comparator groups. | | | | | | | | |
| --- | --- | --- | --- | --- | --- | --- | --- | --- |
| **Study** | **No comparator group^#^** | **Active control group** | **Specific-active control group** | | | | | |
|  |  |  | **CCT vs CDT** | **CCT vs. CAT** | **CCT vs. RT** | **CCT vs Compound** | **CCT vs. Plyometrics** | **CCT vs CCT** |
| Alemdaroglu et al. [1] |  |  | X | X |  |  |  |  |
| Ali et al. [2] |  | X |  |  | X |  |  |  |
| Ali et al. [3] |  | X |  |  |  |  | X |  |
| Alves et al. [4] |  | X |  |  |  |  |  | 1 vs. 2 sessions per week* |
| Arazi et al. [5] |  |  |  |  | X | X | X |  |
| Argus et al. [6] |  |  |  |  |  |  |  | Assisted vs. unresisted vs. resisted ** |
| Argus et al. [7] |  |  |  |  |  |  |  | Strength vs. speed focus |
| Berriel et al. [8] |  |  |  |  |  |  | X |  |
| Biel et al. [9] |  |  |  | X |  |  |  |  |
| Bogdanis et al. [10] |  | X |  |  |  |  |  |  |
| Bogdanis et al. [11] |  |  |  |  |  |  |  | Isometric leg press at different angles (85° vs. 145°) |
| Brito et al. [12] |  | X |  |  | X |  | X |  |
| Cavaco et al. [13] |  | X |  |  |  |  |  | 1 vs. 2 sessions per week* |
| Chakshuraksha et al. [14] |  |  |  |  |  |  |  | Traditional vs. accentuated eccentric load |
| Chatzinikolaou et al. [15] |  | X |  |  |  |  |  |  |
| Dobbs et al. [16] |  |  | X |  |  |  |  |  |
| Dodd et al. [17] |  |  |  |  | X |  | X |  |
| Faude et al. [18] |  | X |  |  |  |  |  |  |
| Freitas et al. [19] |  |  |  |  | X |  |  |  |
| Garcia-Pinillos et al. [20] |  | X |  |  |  |  |  |  |
| Gee et al. [21] |  |  |  |  |  |  |  | CCT vs. reverse contrast training |
| Gonzalez-Rave et al. [22] |  |  |  |  |  |  |  | Athletes vs. non-athletes |
| Hammami et al. [23] |  | X |  |  | X |  |  |  |
| Hammami et al. [24] |  | X |  |  |  |  |  |  |
| Hammami et al. [25] |  | X |  |  |  |  | X |  |
| Hammami et al. [26] |  | X |  |  |  |  |  |  |
| Hammami et al. [27] |  | X |  |  |  |  |  |  |
| Hammami et al. [28] |  | X |  |  |  |  |  |  |
| Juarez et al. [29] |  |  |  |  |  |  |  |  |
| Kobal et al. [30] |  |  | X | X |  |  |  |  |
| Kralova et al. [31] |  | X | X |  |  |  |  |  |
| Kumar et al. [32] |  | X |  |  |  |  |  |  |
| Kumar et al. [33] |  | X |  |  |  |  |  |  |
| Kumar et al. [34] |  | X |  |  |  |  |  | 2 vs. 3 sessions per week (equated volume) |
| Latorre Roman et al. [35] |  | X |  |  |  |  |  |  |
| Li et al. [36] |  | X |  |  | X |  |  |  |
| Li et al. [37] |  |  |  |  | X |  |  |  |
| Liu et al. [38] |  |  |  |  | X |  |  |  |
| MacDonald et al. [39] |  |  |  |  | X |  | X |  |
| McMaster et al. [40] |  |  |  |  |  |  |  | Heavy (60 – 75% 1RM) vs. light (15 – 30% 1RM) ballistic low-load exercise |
| Mesfar et al. [41] |  | X |  |  |  |  |  |  |
| Mihalik et al. [42] |  |  |  |  |  | X |  |  |
| Miranda et al. [43] |  | X |  |  | X |  |  |  |
| Mujika et al. [44] |  | X |  |  |  |  |  |  |
| Nikolic et al. [45] |  | X |  |  |  |  |  |  |
| Pauli et al. [46] |  |  | X |  |  |  |  |  |
| Qiao et al. [47] |  |  |  |  | X |  |  |  |
| Redondo et al. [48] |  | X |  |  |  |  |  |  |
| Schneiker et al. [49] |  |  |  |  | X |  |  |  |
| Scott et al. [50] |  | X |  |  |  |  |  | Traditional vs. variable resistance (23% 1RM from resistance band) method |
| Scott et al. [51] |  | X |  |  |  |  |  | Traditional vs. variable resistance (23% 1RM from resistance band) method |
| Sedano et al. [52] |  | X |  |  | X |  |  |  |
| Shi et al. [53] |  |  |  |  |  |  |  | Traditional vs. variable resistance (40% 1RM using resistance band) method |
| Smilios et al. [54] |  | X |  |  |  |  |  | Heavy load vs. Pmax vs. Pmax + body mass |
| Smith et al. [55] |  | X |  |  |  |  |  | Strength vs. power as high-load activity |
| Spineti et al. [56] |  |  |  |  | X |  |  |  |
| Spineti et al. [57] |  |  |  |  | X |  |  |  |
| Stasinaki et al. [58] |  | X |  |  |  | X |  |  |
| Thapa & Kumar [59] |  |  |  |  |  |  |  | Stronger (relative strength ≥1.75) vs. weaker (relative strength <1.55) |
| Thapa et al. [60] |  | X |  |  |  |  |  |  |
| Thapa et al. [61] |  | X |  |  |  |  |  |  |
| Tsimahidis et al. [62] |  | X |  |  |  |  |  |  |
| Walker et al. [63] | X |  |  |  |  |  |  |  |
| Wallenta et al. [64] |  |  |  |  | X |  |  |  |
| Wang et al. [65] |  |  |  |  |  |  |  | Creatine vs. placebo |
| Watt et al. [66] | X |  |  |  |  |  |  |  |
| Yang et al. [67] |  |  |  |  |  |  |  | Traditional vs. BFR |
| **% of total studies** | **2.9%** | **50%** | **7.4%** | **4.4%** | **26.5%** | **5.9%** | **10.3%** | **26.5%** |
| BFR – blood flow restriction, CCT – complex contrast training, CDT – complex descending training, CAT – complex ascending training, Pmax – load that maximizes mechanical power without accounting for body mass, Pmax + body mass – load that maximizes mechanical power accounting for body mass, RT – resistance training.  ^#^: the study did not include any comparator group (i.e., active or specific-active control group).  *: authors did not report if the total volume was equated.  **: high-load activity was 4RM power clean with lower-load activity with either assisted (using elastic bands), unresisted (only body mass), or resisted (using elastic bands) jumps. | | | | | | | | |

**References**

1. Alemdaroğlu U, Dündar U, Köklü Y, Aşci A, Findikoğlu G. The effect of exercise order incorporating plyometric and resistance training on isokinetic leg strength and vertical jump performance: A comparative study. Isokinetics and Exercise Science. 2013;21(3):211-7.

2. Ali K, Verma S, Ahmad I, Singla D, Saleem M, Hussain ME. Comparison of Complex Versus Contrast Training on Steroid Hormones and Sports Performance in Male Soccer Players. J Chiropr Med. 2019 Jun;18(2):131-8.

3. Ali K, Gupta S, Hussain ME, Alzhrani M, Manzar MD, Khan M, et al. Effect of plyometric versus complex training on core strength, lower limb, and upper limb power in male cricketers: a randomized controlled trial. BMC Sports Science, Medicine and Rehabilitation. 2023 2023/11/27;15(1):160.

4. Alves JM, Rebelo AN, Abrantes C, Sampaio J. Short-term effects of complex and contrast training in soccer players' vertical jump, sprint, and agility abilities. J Strength Cond Res. 2010 Apr;24(4):936-41.

5. Arazi H, Asadi A, Roohi S. Enhancing muscular performance in women: compound versus complex, traditional resistance and plyometric training alone. Journal of Musculoskeletal Research. 2014;17(02):1450007.

6. Argus CK, Gill ND, Keogh JW, Blazevich AJ, Hopkins WG. Kinetic and training comparisons between assisted, resisted, and free countermovement jumps. J Strength Cond Res. 2011 Aug;25(8):2219-27.

7. Argus CK, Gill ND, Keogh JW, McGuigan MR, Hopkins WG. Effects of two contrast training programs on jump performance in rugby union players during a competition phase. Int J Sports Physiol Perform. 2012 Mar;7(1):68-75.

8. Berriel GP, Cardoso AS, Costa RR, Rosa RG, Oliveira HB, Kruel LFM, et al. Does Complex Training Enhance Vertical Jump Performance and Muscle Power in Elite Male Volleyball Players? Int J Sports Physiol Perform. 2022 Apr 1;17(4):586-93.

9. Biel P, Ewertowska P, Stastny P, Krzysztofik M. Effects of Complex Training on Jumping and Change of Direction Performance, and Post-Activation Performance Enhancement Response in Basketball Players. Sports. 2023;11(9):181.

10. Bogdanis GC, Tsoukos A, Brown LE, Selima E, Veligekas P, Spengos K, et al. Muscle Fiber and Performance Changes after Fast Eccentric Complex Training. Med Sci Sports Exerc. 2018 Apr;50(4):729-38.

11. Bogdanis GC, Tsoukos A, Methenitis SK, Selima E, Veligekas P, Terzis G. Effects of low volume isometric leg press complex training at two knee angles on force-angle relationship and rate of force development. Eur J Sport Sci. 2019 Apr;19(3):345-53.

12. Brito J, Vasconcellos F, Oliveira J, Krustrup P, Rebelo A. Short-term performance effects of three different low-volume strength-training programmes in college male soccer players. J Hum Kinet. 2014 Mar 27;40:121-8.

13. Cavaco B, Sousa N, Dos Reis VM, Garrido N, Saavedra F, Mendes R, et al. Short-term effects of complex training on agility with the ball, speed, efficiency of crossing and shooting in youth soccer players. J Hum Kinet. 2014 Sep 29;43:105-12.

14. Chakshuraksha P, Apanukul S. Effects of Accentuated Eccentric Loading Combined with Plyometric Training on Strength, Power, Speed, and Agility in Male Rugby Players. Journal of Exercise Physiology Online. 2021;24(3).

15. Chatzinikolaou A, Michaloglou K, Avloniti A, Leontsini D, Deli CK, Vlachopoulos D, et al. The Trainability of Adolescent Soccer Players to Brief Periodized Complex Training. Int J Sports Physiol Perform. 2018 May 1;13(5):645-55.

16. Dobbs CW, Gill ND, Smart DJ, McGuigan MR. The training effect of short term enhancement from complex pairing on horizontal and vertical countermovement and drop jump performance. J Strength Cond Res. 2015 Feb 3.

17. Dodd DJ, Alvar BA. Analysis of acute explosive training modalities to improve lower-body power in baseball players. J Strength Cond Res. 2007 Nov;21(4):1177-82.

18. Faude O, Roth R, Di Giovine D, Zahner L, Donath L. Combined strength and power training in high-level amateur football during the competitive season: a randomised-controlled trial. J Sports Sci. 2013;31(13):1460-7.

19. Freitas TT, Calleja-González J, Carlos-Vivas J, Marín-Cascales E, Alcaraz PE. Short-term optimal load training vs a modified complex training in semi-professional basketball players. J Sports Sci. 2019 Feb;37(4):434-42.

20. García-Pinillos F, Martínez-Amat A, Hita-Contreras F, Martínez-López EJ, Latorre-Román PA. Effects of a contrast training program without external load on vertical jump, kicking speed, sprint, and agility of young soccer players. J Strength Cond Res. 2014 Sep;28(9):2452-60.

21. Gee TI, Harsley P, Bishop DC. Effect of 10 Weeks of Complex Training on Speed and Power in Academy Soccer Players. Int J Sports Physiol Perform. 2021 Aug 1;16(8):1134–9.

22. González-Ravé JM, Delgado M, Vaquero M, Juarez D, Newton RU. Changes in vertical jump height, anthropometric characteristics, and biochemical parameters after contrast training in master athletes and physically active older people. J Strength Cond Res. 2011 Jul;25(7):1866-78.

23. Hammami M, Negra Y, Shephard RJ, Chelly MS. The Effect of Standard Strength vs. Contrast Strength Training on the Development of Sprint, Agility, Repeated Change of Direction, and Jump in Junior Male Soccer Players. J Strength Cond Res. 2017 Apr;31(4):901-12.

24. Hammami M, Negra Y, Shephard RJ, Chelly MS. Effects of leg contrast strength training on sprint, agility and repeated change of direction performance in male soccer players. J Sports Med Phys Fitness. 2017 Nov;57(11):1424-31.

25. Hammami M, Gaamouri N, Shephard RJ, Chelly MS. Effects of Contrast Strength vs. Plyometric Training on Lower-Limb Explosive Performance, Ability to Change Direction and Neuromuscular Adaptation in Soccer Players. J Strength Cond Res. 2019 Aug;33(8):2094-103.

26. Hammami M, Gaamouri N, Aloui G, Shephard RJ, Chelly MS. Effects of a Complex Strength-Training Program on Athletic Performance of Junior Female Handball Players. Int J Sports Physiol Perform. 2019 Feb 1;14(2):163-9.

27. Hammami M, Gaamouri N, Cherni Y, Gaied S, Chelly MS, Hill L, et al. Effects of complex strength training with elastic band program on repeated change of direction in young female handball players: Randomized control trial. International Journal of Sports Science & Coaching. 2022;17(6):1396-407.

28. Hammami M, Gaamouri N, Cherni Y, Chelly MS, Hill L, Knechtle B. Effects of contrast strength training with elastic band program on sprint, jump, strength, balance and repeated change of direction in young female handball players. International Journal of Sports Science & Coaching. 2022;17(5):1147-57.

29. Juárez D, González-Ravé JM, Navarro F. Effects of complex vs non complex training programs on lower body maximum strength and power. Isokinetics and Exercise Science. 2009;17(4):233-41.

30. Kobal R, Loturco I, Barroso R, Gil S, Cuniyochi R, Ugrinowitsch C, et al. Effects of Different Combinations of Strength, Power, and Plyometric Training on the Physical Performance of Elite Young Soccer Players. J Strength Cond Res. 2017 Jun;31(6):1468-76.

31. Králová T, Hammerová T, Vanderka M, Cacek J, Bozděch M, Vrbas V. The Effect of 8 Weeks of Complex Training Methods on the Countermovement Jump Performance. Studia sportiva. 2020;14(2):8-17.

32. Kumar G, Pandey V. Effect of Complex Training on Aerobic and Anaerobic Power of Amateur Athletes. Physical Education Theory and Methodology. 2023;23(1):65-71.

33. Kumar G, Pandey V, Ramirez-Campillo R, Thapa RK. Effects of Six-Week Pre-Season Complex Contrast Training Intervention on Male Soccer Players’ Athletic Performance. Polish Journal of Sport and Tourism. 2023;30(3):29-35.

34. Kumar G, Pandey V, Thapa RK, Weldon A, Granacher U, Ramirez-Campillo R. Effects of Exercise Frequency with Complex Contrast Training on Measures of Physical Fitness in Active Adult Males. Sports (Basel). 2023 Jan 5;11(1).

35. Latorre Román P, Villar Macias FJ, García Pinillos F. Effects of a contrast training programme on jumping, sprinting and agility performance of prepubertal basketball players. J Sports Sci. 2018 Apr;36(7):802-8.

36. Li F, Wang R, Newton RU, Sutton D, Shi Y, Ding H. Effects of complex training versus heavy resistance training on neuromuscular adaptation, running economy and 5-km performance in well-trained distance runners. PeerJ. 2019;7:e6787.

37. Li F, Nassis GP, Shi Y, Han G, Zhang X, Gao B, et al. Concurrent complex and endurance training for recreational marathon runners: Effects on neuromuscular and running performance. Eur J Sport Sci. 2021 Sep;21(9):1243-53.

38. Liu M, Zhou K, Li B, Guo Z, Chen Y, Miao G, et al. Effect of 12 weeks of complex training on occupational activities, strength, and power in professional firefighters. Front Physiol. 2022;13:962546.

39. MacDonald CJ, Lamont HS, Garner JC. A comparison of the effects of 6 weeks of traditional resistance training, plyometric training, and complex training on measures of strength and anthropometrics. J Strength Cond Res. 2012 Feb;26(2):422-31.

40. McMaster D, Gill N, McGuigan M, Cronin J. Effects of complex strength and ballistic training on maximum strength, sprint ability and force-velocity-power profiles of semi-professional rugby union players. J Aust Strength Cond. 2014;22(1):17-30.

41. Mesfar A, Hammami R, Selmi W, Gaied-Chortane S, Duncan M, Bowman TG, et al. Effects of 8-Week In-Season Contrast Strength Training Program on Measures of Athletic Performance and Lower-Limb Asymmetry in Male Youth Volleyball Players. Int J Environ Res Public Health. 2022 May 27;19(11).

42. Mihalik JP, Libby JJ, Battaglini CL, McMurray RG. Comparing short-term complex and compound training programs on vertical jump height and power output. J Strength Cond Res. 2008 Jan;22(1):47-53.

43. Miranda C, Rago V, Silva JR, Rebelo A. Effects of traditional vs. complex strength training added to regular football training on physical capacities in U19 football players: a team study. Sport Sciences for Health. 2021:1-10.

44. Mujika I, Santisteban J, Castagna C. In-season effect of short-term sprint and power training programs on elite junior soccer players. J Strength Cond Res. 2009 Dec;23(9):2581-7.

45. Nikolic D, Beric D, Kocic M, Daskalovski B. Complex training and sprint abilities of young basketball players. Facta Universitatis, Series: Physical Education and Sport. 2017;15(1):025-36.

46. Pauli PH, de Borba EF, da Silva MP, Martins MVS, Batista MM, Tartaruga MP. Effects of Complex and Contrast Training on Strength, Power, and Agility in Professional Futsal Players: A Preliminary Study. Journal of Science in Sport and Exercise. 2023 2023/08/02.

47. Qiao Z, Guo Z, Li B, Liu M, Miao G, Zhou L, et al. The effects of 8-week complex training on lower-limb strength and power of Chinese elite female modern pentathlon athletes. Front Psychol. 2022;13:977882.

48. Redondo JC, Alonso CJ, Sedano S, de Benito AM. Effects of a 12-week strength training program on experimented fencers' movement time. J Strength Cond Res. 2014 Dec;28(12):3375-84.

49. Schneiker KT, Fyfe JJ, Teo SYM, Bishop DJ. Comparative Effects of Contrast Training and Progressive Resistance Training on Strength and Power-Related Measures in Subelite Australian Rules Football Players. J Strength Cond Res. 2023 Jan 19.

50. Scott DJ, Ditroilo M, Orange ST, Marshall P. The Effect of Complex Training on Physical Performance in Rugby League Players. Int J Sports Physiol Perform. 2023 Mar 1;18(3):240-7.

51. Scott DJ, Marshall P, Orange ST, Ditroilo M. The Effect of Complex Training on Muscle Architecture in Rugby League Players. Int J Sports Physiol Perform. 2023 Mar 1;18(3):231-9.

52. Sedano S, Marín PJ, Cuadrado G, Redondo JC. Concurrent training in elite male runners: the influence of strength versus muscular endurance training on performance outcomes. J Strength Cond Res. 2013 Sep;27(9):2433-43.

53. Shi L, Lyons M, Duncan M, Chen S, Chen Z, Guo W, et al. Effects of Variable Resistance Training Within Complex Training on Neuromuscular Adaptations in Collegiate Basketball Players. J Hum Kinet. 2022 Oct;84:174-83.

54. Smilios I, Sotiropoulos K, Christou M, Douda H, Spaias A, Tokmakidis SP. Maximum power training load determination and its effects on load-power relationship, maximum strength, and vertical jump performance. J Strength Cond Res. 2013 May;27(5):1223-33.

55. Smith CE, Lyons B, Hannon JC. A pilot study involving the effect of two different complex training protocols on lower body power. Human Movement. 2014;15(3):141-6.

56. Spineti J, Figueiredo T, Bastos DEOV, Assis M, Fernandes DEOL, Miranda H, et al. Comparison between traditional strength training and complex contrast training on repeated sprint ability and muscle architecture in elite soccer players. J Sports Med Phys Fitness. 2016 Nov;56(11):1269-78.

57. Spineti J, Figueiredo T, Willardson J, Bastos de Oliveira V, Assis M, Fernandes de Oliveira L, et al. Comparison between traditional strength training and complex contrast training on soccer players. J Sports Med Phys Fitness. 2019 Jan;59(1):42-9.

58. Stasinaki AN, Gloumis G, Spengos K, Blazevich AJ, Zaras N, Georgiadis G, et al. Muscle Strength, Power, and Morphologic Adaptations After 6 Weeks of Compound vs. Complex Training in Healthy Men. J Strength Cond Res. 2015 Sep;29(9):2559-69.

59. Thapa RK, Kumar G. Does complex contrast training induce higher physical fitness improvement in stronger compared to weaker individuals? Montenegrin Journal of Sports Science and Medicine. 2023;12(1):Ahead of Print.

60. Thapa RK, Kumar G, Weldon A, Moran J, Chaabene H, Ramirez-Campillo R. Effects of complex-contrast training on physical fitness in male field hockey athletes. Biomedical Human Kinetics. 2023;15(1):201-10.

61. Thapa RK, Kumar G, Raizada S, Bagchi A. Effects of Contrast Training with Two Sessions Weekly Frequency on Physical Fitness of University-Level Male Soccer Players. Physical Education Theory and Methodology. 2023;23(6):886-93.

62. Tsimahidis K, Galazoulas C, Skoufas D, Papaiakovou G, Bassa E, Patikas D, et al. The effect of sprinting after each set of heavy resistance training on the running speed and jumping performance of young basketball players. J Strength Cond Res. 2010 Aug;24(8):2102-8.

63. Walker S, Ahtiainen JP, Häkkinen K. Acute neuromuscular and hormonal responses during contrast loading: effect of 11 weeks of contrast training. Scand J Med Sci Sports. 2010 Apr;20(2):226-34.

64. Wallenta C, Granacher U, Lesinski M, Schünemann C, Muehlbauer T. Effects of complex versus block strength training in athletic performance of elite youth soccer players. Sportverletzung· Sportschaden. 2016;30(01):31-7.

65. Wang CC, Fang CC, Lee YH, Yang MT, Chan KH. Effects of 4-Week Creatine Supplementation Combined with Complex Training on Muscle Damage and Sport Performance. Nutrients. 2018 Nov 2;10(11).

66. Watts DG, Kelly VG, Young KP. The efficacy of a four-week intervention of complex training on power development in elite junior volleyball players. J Aust Strength Cond. 2012;20(2):12-22.

67. Yang S, Zhang P, Sevilla-Sanchez M, Zhou D, Cao J, He J, et al. Low-Load Blood Flow Restriction Squat as Conditioning Activity Within a Contrast Training Sequence in High-Level Preadolescent Trampoline Gymnasts. Front Physiol. 2022;13:852693.
